# Specialty Grand Challenge Article- Social Neuroergonomics

**DOI:** 10.3389/fnrgo.2021.654597

**Published:** 2021-04-07

**Authors:** Frank Krueger, Eva Wiese

**Affiliations:** ^1^School of Systems Biology, George Mason University, Fairfax, VA, United States; ^2^Institute of Psychology and Ergonomics, Berlin, Germany

**Keywords:** from automation to autonomy, from observation to interaction, from explicit to implicit measures, from dyads to groups, from laboratory to natural environments

## Introduction

Social Neuroergonomics (SNE) is a transdisciplinary field (including psychology, human factors, engineering, social neuroscience) devoted to the application of knowledge of the neurobiological underpinnings of social processes and behaviors (ranging from neural to hormonal to cellular and genetic mechanisms) to the design, engineering, and evaluation of human-machine systems. The tremendous advances that have been accomplished in each of these fields can inspire data-driven hypotheses, foster experimental designs, and increase neuroergonomic theories' relevance, which is not achievable from only a single-field perspective. Based on a profound understanding of both the social nature of human brains and the principles of human-centered design, SNE has the unique potential to advance our understanding of the psychoneurobiological basis of whether and how humans engage in social interactions with technology (ranging from automated technical systems to autonomous robots) and to use these insights to foster more efficient and satisfying human-machine interactions (HMIs) (e.g., reduction of human errors, increase of trust and productivity, enhancement of safety) in everyday settings (Hancock et al., 2011). Like any emerging research field, SNE will face challenges in the upcoming years that have to be acknowledged, addressed, and resolved. This *Special Grand Challenge* highlights some of these challenges to ensure the success of SNE as a multi-level integrative field with high relevance and impact on our everyday lives as well as practical implications in diverse sectors such as healthcare, education, therapy, and entertainment (Parasuraman, 2011; Ayaz and Dehais, 2019).

## Challenge 1: From Automation to Autonomy

One challenge for SNE will be to optimally foster the shift from automated (i.e., executing a pre-defined task by rule-based responses in reasonably well-known and structured environments) to fully autonomous (i.e., executing a task by adaptive-based learning or artificial intelligence-based capabilities in unknown and changing environments) systems, such as embodied social robots, that need to be adaptive, personified, independent, socially intelligent, and indeterminate to satisfy users' needs (Schaefer et al., 2016). Our future will inevitably be shaped by a co-existence of humans as social, biological agents and social, artificially intelligent machine agents. Social embodied robots are already increasingly taking on numerous roles in our society in various domains, which highlights the necessity of a scientific investigation of the psychoneurobiological basis of HMIs as well as the formulation of empirically derived guidelines for the design and evaluation of such systems (Fujitam, 2004). However, we are still confronted with the reality that current social robot systems fall short of expectations humans have about intuitive, efficient, and rewarding mutual interactions (Dautenhahn et al., 2002). One critical psychological factor for successful social interactions is that we are more motivated to engage in interactions with entities that are believed “to have a mind of their own” —with internal states like intentions, emotions, or preferences (i.e., mind perception, Gray et al., 2007). Mind perception is an attributional process by which a *perceiver* imbues a human-like mind status to a *perceived* agent based on certain features of the perceiver (e.g., state and/or trait loneliness, knowledge about technology), as well as certain features of the perceived (e.g., human appearance, biological motion, similarity-to-self, Waytz et al., 2010). Once the mind is attributed to agents—human or non-human—their behaviors and actions tend to be interpreted in mentalistic terms (i.e., driven by human-like internal states) and interacting with such agents triggers motivational, affective, and cognitive processes in our social brains (Wiese et al., 2017). However, anthropomorphic appearance does not always lead to more social human-robot interactions, as subtle imperfections of robots that physically appear extremely—but not perfectly—human-like (e.g., androids, geminoids) can become disturbing and hinder social HMIs (i.e., “uncanny valley” hypothesis, Mori, 1970). Over the last decades, social neuroscience has shed some light on which perceptual-motor features activate social brain areas (e.g., goal-directed actions for the mirror-neuron system) during human-human interactions; building upon these insights, a minimal set of design features can be determined to evoke similar processes during HMIs (Yamaoka et al., 2007)—harvesting behavioral, psychological, and neurobiological methodologies. Therefore, to facilitate the adjustment for the transition from automated to fully autonomous social agents, SNE can help us to formulate a minimal set of design features triggering social brain networks in a diverse set of users to develop social robots that maximize the likelihood of human engagement in satisfying social interactions with these agents in everyday environments (Fong et al., 2003).

## Challenge 2: From Observation to Interaction

Another challenge for the growing field of SNE is to shed light on the multifaceted foundation of social cognition during HMIs utilizing ecologically-sound experimental paradigms that investigate dynamically unfolding interactions over time instead of static observations of the social situation. So far, a plethora of studies dominantly examine the brain in isolation by employing social observation paradigms instead of imposing interactive paradigms where social agents adopt complementary and alternating roles during the progression of social interactions that affect immediate and upcoming occurrences between humans and robotic agents. A “second-person” neuroscientific framework (Schilbach et al., 2013) or “two-person neuroscience” approach (Hari and Kujala, 2009) for SNE is needed for which neural mechanisms of the human's brain are evaluated during real-time reciprocal social interactions—imposing either turn-based or mutual realistic HMIs. Social interactions are more than just tapping into an agent's social knowledge representations; instead, they initiate shared processing and knowledge between agents (Redcay and Schilbach, 2019). Neuroscience has proven that variations in the configuration of brain networks exist when we are interacting in real-time with other agents (i.e., “online” social cognition mirroring the dynamics between agents) in comparison to observing other agents (i.e., “offline” social cognition mirroring the statics inside the agent) (Pfeiffer et al., 2013). Overall, the neural mechanisms of dynamic real-time social HMI are largely unexplored and can be considered the “dark matter” in studying HMI (Schilbach et al., 2013). To close this gap, SNE should develop, test, and validate empirical paradigms that allow the measurement of neuropsychological indices employing hyperscanning techniques in real-time dynamic social HMI interactions. These measurements should not be confined to short one-time interactions between humans and expensive, difficult-to-handle robot platforms in research laboratories, instead should be obtained in everyday environments with easy-to-use social robot platforms (e.g., Cozmo) over longer periods. To make long-term social interactions with robots motivating to the general public, SNE will need input from computer science to develop social artificially intelligent systems that can attune to human input and adapt flexibly in dynamic interactions.

## Challenge 3: From Explicit to Implicit Measures

Another challenge is to widen the spectrum of examining psychological processes from explicit (occurring controlled and slow with conscious access and control) to implicit (occurring automatically and fast without conscious awareness or control). If studying social interaction (as opposed to observation) is seen as the “dark matter” of SNE, then investigating implicit social processes (as opposed to explicit processes) can be regarded the “dark energy” of SNE. Like dark energy is taking up most of our universe, implicit processes shape most of our social interactions. As two levels of social cognition, implicit and explicit processes often interplay and influence social perception, cognition, and interaction—sometimes complementary and other times oppositional (Frith and Frith, 2008). Studying how brain networks recruited for implicit processes (e.g., salience network) interact with brain networks recruited for explicit processes (e.g., central-executive network) (Forbes and Grafman, 2013) can provide us with valuable insights on how to understand better and design mutual, dynamic HMI. Despite the fast growth in the field of HMI, reliable and innovative implicit measures predicting outcomes in HMI (e.g., social bonding, joint performance) are missing. Most studies employ primarily self-report or measures of explicit processes (e.g., subjective ratings) to evaluate trust in HMIs (Sanders et al., 2016). However, research has shown that assessing measures of implicit attitudes toward machines (e.g., via Implicit Association Test) can be more predictive of trust behavior than objective measures of explicit processes (Merritt et al., 2013). In this vein, it will be important for SNE to advance the field of social human-technology interaction by proposing innovative paradigms that combine measures of implicit and explicit processes with neuroscience methods to ultimately develop empirically derived theories of when and how critical operative social processes shape dynamic HMI.

## Challenge 4: From Dyads to Groups

Another challenge is to broaden the research focus on studying HMIs ranging from dyads over groups to cultures and their underpinning neural mechanisms to understand better how these psychoneurobiological processes contribute to more beneficial HMIs. On the one hand, dyads are different from groups: dyads can both form and dissolve faster, dyads experience stronger and sometimes different emotions than people in groups, and dyads are less complex than groups as they lack group-specific manifestations (e.g., socialization, coalition formation, and majority/minority influence) (Moreland, 2010). Robots are most commonly expected to assist groups of people, such as in public places (e.g., shopping malls, airports, museums) (Burgard et al., 1999). However, to date, little is known about the environmental and psychological (i.e., motivational, affective, and cognitive) factors that foster social integration, group formation, and interaction within mixed human-robot groups—as current research primarily focuses on dyadic interactions with social agents. On the other hand, we often assume that our research findings are universal, but comparative studies report differences, for example, between industrialized and small-scale societies and Western and non-Western cultures. However, most participants in neuroergonomic studies are from Western, Educated, Industrialized, Rich, and Democratic (WEIRD) populations; therefore, the question of the representativeness of those results should be kept in mind (Falk et al., 2013). For example, various cultures exhibit substantial disparities in cognitive (e.g., perception, categorization, deduction) and social (e.g., fairness, cooperation, morality) mechanisms (Henrich et al., 2010). Furthermore, differences in social interactions due to dyadic, group, and cultural variations evoke differential psychological processes that influence neural processing, and recent neuroimaging experiments have highlighted the neural networks underlying those variances (Ames and Fiske, 2010). Therefore, the next phase of SNE research should focus on expanding neuroergonomic research to groups (besides dyads) and diverse cultural populations (besides WEIRD populations) (Kedia et al., 2017)—studying the neurophysiological correlates of human-machine/human-AI teaming in comparision to human-human teaming by utilizing hyperscanning techniques.

## Challenge 5: From Laboratory to Natural Environments

A final challenge for SNE is implementing a systematic experimental approach to develop interactive human-robot paradigms and procedures by starting in the laboratory and adjusting them for field and finally natural environments combining psychobehavioral with neurobiological measures. Laboratory experiments control independent variables under highly standardized conditions; field experiments are performed in an everyday environment but still manipulate independent variables; whereas natural experiments are conducted with no control over independent variables as they occur naturally in real life. Applying concurrent neuroimaging techniques—combining functional magnetic resonance imaging (fMRI) with functional near-infrared spectroscopy (fNIRS) or electroencephalography (EEG) to optimize spatial and temporal resolution—while comparing dynamic HMI with human-human interactions under laboratory conditions will enable us to identify target regions within social networks for subsequent field and natural experiments. Most neuroscience methods such as fMRI impose heavy constraints on and exhaust participants (e.g., constrainment of movements, awareness of being observed, multiplicity of trials within the same task) that results in biases of psychological processes and prevent them from reacting naturally as in real-world situations (Kedia et al., 2017). Identified regions within necessary social networks via fMRI can be later targeted with mobile and portable functional neuroimaging tools (e.g., fNIRS, EEG) in field or natural settings to explore mutual moment-to-moment dynamic HMI in more ecological environments (e.g., development of HR relationships and attachment, Krueger et al., 2021). These methodological developments should be combined with a wider use and creation of analysis methods (e.g., computational methods) to capture inter-individual variations at the structural and functional brain level during real-world HMI. SNE should concentrate on the problem of the generalizability and reproducibility of research findings—including under-powered neuroimaging experiments, dependency on flexible statistical analyses, and *post-hoc* modification of hypotheses—which can lead to a distorted characterization of brain activity. Such challenges have to be addressed to avoid a replication crisis and before making any claim regarding practical applications. Moreover, to implement HMI studies “in the wild,” researchers need to identify social robot platforms that are easy-to-use, affordable, programmable, and AI-compatible (e.g., Cozmo) and explore their utility for the empirical investigation of HMI (including code-sharing on open science platforms or platforms like Github) in everyday environments. In the same vein, SNE should also explore the utility of neurophysiological measurement tools that are user-friendly and can be used by non-scientists at home (e.g., Muse headband).

## Conclusion

While SNE has started to illuminate the behavioral, psychological, and neurobiological processes of HMIs, this newly emerging research area is still at its inception. However, the identified five challenges—development from automatic to autonomous systems, the progress from observation to interaction, the expansion from extrinsic to intrinsic measures, the extension from dyads to groups, and the move from the laboratory to natural experiments—will allow both conceptually and methodologically ground-breaking insights that point to a bright future for this field. With the overlapping and similarly rapidly developing sister fields of neuroergonomics and other related fields, SNE holds a great deal of promise in discovering the neurobiological processes that underlie people's emotions, feelings, and behaviors to enhance current and future HMIs, especially in daily life and natural environments. We hope that the outlined grand challenges will lead neuroscientists, psychologists, and engineers to work together more consistently, with a shared assumption that the comprehension of HMIs will be improved by an integrative analysis that incorporates levels of investigations spanning from genes over brains to behaviors and cultures. Moreover, we expect that SNE will attract the attention of researchers, professionals, and the public, who are increasingly building on SNE's insights and methodologies to explore HMIs successfully in the future. Eventually, understanding HMIs will help us to understand ourselves better.

## Author Contributions

FK and EW handled the entirety of this paper.

## Conflict of Interest

The authors declare that the research was conducted in the absence of any commercial or financial relationships that could be construed as a potential conflict of interest.
